# Ring bound states in the continuum with broken time-reversal symmetry

**DOI:** 10.1016/j.isci.2025.114103

**Published:** 2025-11-17

**Authors:** Yun-tuan Fang, Fan Bu, Sailing He

**Affiliations:** 1 School of Computer Science and Communication Engineering, Jiangsu University, Zhenjiang 212013, P.R. China; 2 Department of Electromagnetic Engineering, School of Electrical Engineering, Royal Institute of Technology, 100 44 Stockholm, Sweden; 3 Centre for Optical and Electromagnetic Research, National Engineering Research Center for Optical Instruments, State Key Laboratory of Modern Optical Instrumentation, Zhejiang Provincial Key Laboratory for Sensing Technologies, Zhejiang University, Hangzhou 310058, China

**Keywords:** Physics, Photonics, Applied sciences

## Abstract

Bound states in the continuum (BICs) are general discrete mode points that typically have narrow-angle responses. To break this limitation on the performance of BICs, we propose a magneto-optical photonic crystal slab and study its eigen modes and far-field polarizations. Unlike general BICs, we find a continuous distribution with a ring shape for the quasi-BICs in *k*-space and analyze the forming mechanism. The ring BICs provide a special response of spatial directions and can be modulated by multiple structural parameters. Different from the linear polarizations of far-field from general BICs, the far-field polarizations within the ring qBICs are either ideally circular polarization or elliptical polarizations with all potential values of ellipticities leading to infinite density of states. The ring qBICs allow us to manipulate the polarization with more degrees of freedom for potential applications.

## Highlights

• The ring qBICs are formed from a photonic crystal slab under the magneto-optical effect
• The polarization states undergo continuous evolution in the ring qBICs
• The circular polarizations occur in a large range of k space

## Graphical abstract

**Figure:**
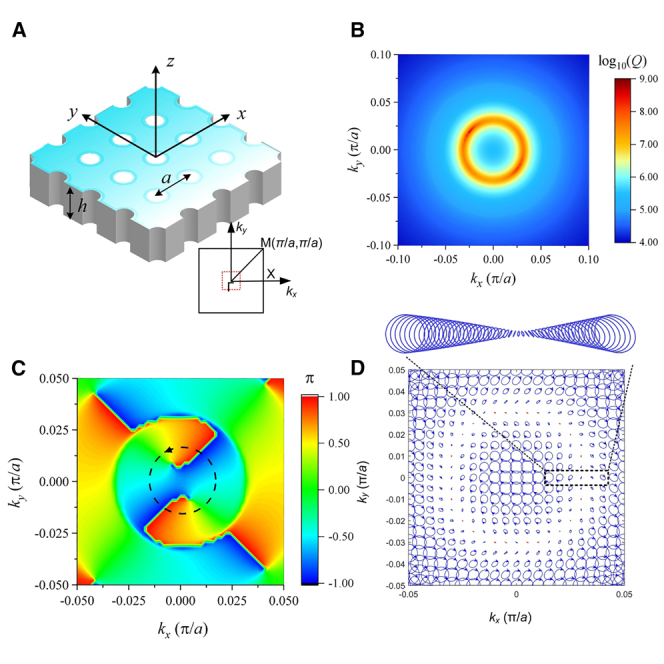

## Introduction

Resonance is the source of many interesting physical phenomena. An important factor for highly efficient functional devices from resonance structures is the quality (*Q*) factor of their resonant modes. Photonic bound states in the continuum (BICs) allow optical resonance modes with ultra-high Q-factors, making them excellent candidates for creating exotic light-matter interactions.^1^^,^^2^^,^^3^^,^^4^^,^^5^^,^^6^^,^^7^ For instance, BICs have facilitated advancements such as lasers,^8^^,^^9^ sensors,^10^^,^^11^ light absorption,^12^ enhancement of harmonic generation^13^^,^^14^^,^^15^^,^^16^ and unidirectional lasing and wavefront transformation.^17^ Various structures and mechanisms have been proposed to achieve BICs, including symmetry-protected BIC,^6^^,^^7^ and the Friedrich-Wintgen (FW) BIC.^18^^,^^19^^,^^20^ FW BIC arises from the interference of resonances belonging to different channels. When two resonances pass each other as a function of a continuous parameter, their coupling will cause an avoided crossing of the resonance dispersion curves. At a specific value of the tuning parameter, the width of one resonance vanishes, and hence it becomes a BIC. The formation of BICs is usually due to multiple mechanisms. Despite the significant advances achieved so far in photonic BICs, some limitations still exist in practical applications. When modes deviate from BIC, their *Q* values undergo rapid attenuation. This limitation imposes constraints on the performance of BICs when subjected to a wide-angle illumination. To obtain the high *Q*-factors in a wider range of *k*-space, researchers have merged multiple BICs in PhC slabs.^21^^,^^22^^,^^23^ Merging BICs can enhance the *Q* factor of all nearby resonances in the same band, but the expanded high-Q range is still limited. Other researchers have achieved wide-angle responses in photonic BICs through the construction of moiré BICs in one-dimensional PhC slabs.^7^ However, due to the one-dimensional structure, the wide angles are limited in one direction. Major limitations on BIC application result from the fact that all types of BICs are discrete modes at Γ or high-symmetry points in *k*-space.^22^ Therefore, if we can construct BICs in a continuous *k*-space, the constraints on BICs can be largely relieved.

Ref.^24^ attributes the nature of BICs in PhC slabs to the topological charges corresponding to far-field polarization singularities. However, the deeper origin of BICs in such PC slabs is because the structures satisfy the conditions of both spatial and time-reversal symmetries. The ideal BICs have infinite Q factor and can hardly be excited through the external source, which limits their practical application. To overcome this limitation, researchers usually break the structure symmetry to transform the BICs into quasi (Q)-BICs or other modes. In fact, many researchers have just studied the evolution of BIC through the breaking of spatial symmetry.^25^^,^^26^^,^^27^^,^^28^^,^^29^ By breaking in-plane inversion (C_2_) symmetry, pairs of circularly polarized states could spawn from the eliminated bound states in the continuum.^25^ Breaking up-down symmetry can lead to unidirectional guided resonance.^26^ Breaking both in-plane and out-of-plane symmetries results in an intrinsic chiral response.^27^ In another work, the breaking of simultaneously in-plane and out-of-plane symmetries has achieved the efficient and controllable emission of circularly polarized light from resonant metasurfaces based on the physics of chiral quasi-bound states in the continuum.^28^ Through breaking the symmetry of the individual resonators in the array, the measured transmission spectrum of the metasurfaces shows a complete evolution from a resonant peak to a resonant dip.^29^ All the above works have only considered the breaking of spatial symmetry, but the formation of BIC in PhC slabs is usually based on both spatial and time symmetries. An important question is, can BICs exist in the PC slabs that keep spatial symmetry but break time-reversal symmetry? BICs in PC slabs are usually vortex singularities (V points) in the polarization vector field of far-field radiation. The simultaneous spatial and time-reversal symmetry requires that the polarization vectors around V points are linear.^24^ Thus, another question arises. If BIC still occurs with broken time-reversal symmetry, are the polarization vectors around BIC still linear? Here, we firstly show that broken time-reversal symmetry enables circular and elliptical polarizations.

## Results

### Design of the model and appearance of the two Q-peaks

**Figure 1:**
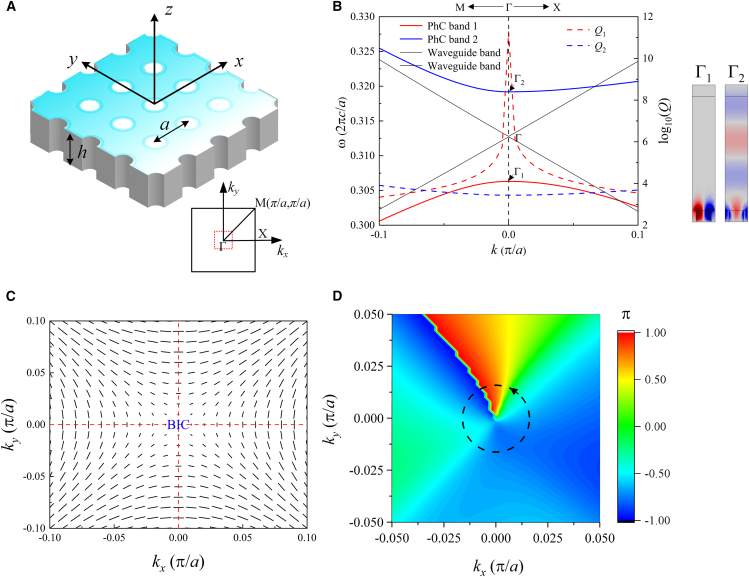
Structure and mode analysis (A) Schematic diagram of the model. Inset: The first Brillouin zone. (B) Frequency bands on the left *y* axis and the *Q* values on the right *y* axis. The two crossing straight lines are the two waveguide bands. (C) The far-field polarization distribution for band 1 with the BIC indicated. (D) The phase distribution of the far-field polarizations for band 1.

We report a two-dimensional (2D) PhC slab that supports the FW BICs. We start by considering a PC slab that is periodic in *x* and *y* and has up-down symmetry (σ_z_). The PhC slab is made of a gyromagnetic material yttrium-iron-garnet (YIG) slab (with permittivity 15.6) with air holes forming a square lattice with lattice constant *a*, which is schematically depicted in Figure 1A with the inset of the first Brillouin zone. The slab thickness is *h* = *a*. The band structure of the quasi-TM modes is calculated by the COMSOL eigenfrequency solver. For the structure slab without the external magnetic field and without holes, two typical crossing plane waveguide bands are obtained in Figure 1B. There are two degenerate guide modes at the crossing point are in the two opposite directions. When the slab is made into a 2D PhC with in-plane inversion symmetry, the corresponding two anti-crossing bands (with serial numbers 1 and 2), which evolved from plane waveguide bands, are also shown in Figure 1B. The two bands are obtained through scanning *k* along the Μ-Γ-Χ direction. The formula *Q* = *ω*_0_/2*γ* is used to calculate the quality factor distribution shown in the figure. Here, *ω*_0_ and *γ* are the real and imaginary parts of the intrinsic frequency, respectively, and the imaginary part is related to the radiation loss. The lifted modes, Γ_1_ with a *Q* peak on band 1 and Γ_2_ with the *Q* dip on band 2, are BIC and leaky mode (resonance mode), respectively. The structure periodicity results in the coupling of guided modes to the continuum of propagating modes in the surrounding free space. The strong coupling induces constructive and destructive interference. The former produces a leaky mode Γ_2_ with strong radiation losses; the latter leads to a BIC Γ_1_with an infinite radiative *Q* factor. The generation mechanism of the two modes is based on the time-domain coupled mode theory (TCMT), which is derived and discussed in method details. The formation of BICs is usually due to multiple mechanisms. The eigen mode fields *E*_z_ of Γ_1_ and Γ_2_ in the inset of Figure 1B have odd and even parities, respectively. The mode Γ_1_ cannot be matched with the radiation plane waves, so it is a symmetry-protected BIC. In view of the topological nature of BIC, because of the simultaneously spatial and time-reversal symmetries, the far-field polarizations of band 1 are linear and plotted in Figure 1C through scanning *k*_*x*_ and *k*_*y*_ from −0.1 *π*/*a* to 0.1 *π*/*a*. The corresponding distribution of phases (the azimuthal angles *ϕ* of polarization vectors) is also plotted in Figure 1D. Γ_1_ is a singular point of polarizations at which the polarization direction cannot be defined. The topological charge can be defined as^24^

(Equation 1) $$q=\frac{1}{2\pi }\oint dk\cdot \nabla_{k}\phi \left(k\right)$$

The integration is performed counterclockwise along any closed curve around the Γ_1_ point. The topological charge of band 1 is *q* = 1.

**Figure 2:**
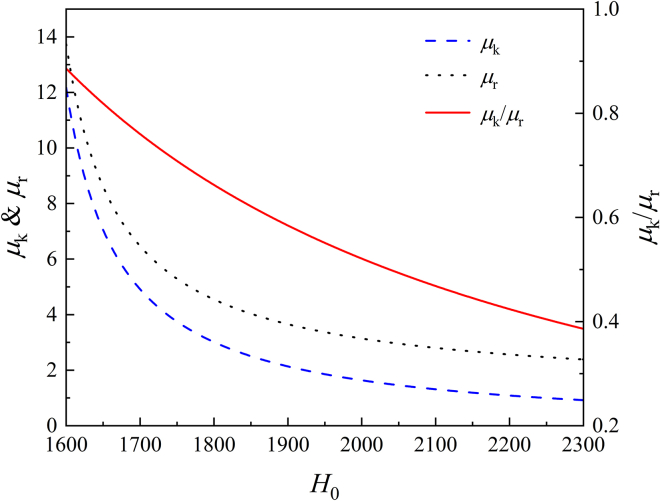
Dependence of *μ*_*r*_ and *μ*_*κ*_ as well as *μ*_*κ*_/*μ*_*r*_ on the applied bias magnetic field *H*_0_

What will happen to the bands and modes Γ_1_ and Γ_2_ when the magnetic field is applied to the structure? Under the action of an applied bias magnetic field *H*_0_ along the *z* direction, the magnetic permeability of the YIG material $\tilde{\mu }$ is a tensor, and it satisfies the following form:^30^

(Equation 2) $$\tilde{\mu }=\left[\begin{array}{ccc} \mu_{r} & i\mu_{\kappa } & 0\\ -i\mu_{\kappa } & \mu_{r} & 0\\ 0 & 0 & \mu_{0} \end{array}\right]$$

where $\mu_{r}=\mu_{0}\left(1+\frac{\omega_{0}\omega_{m}}{\omega_{0}^{2}-\omega^{2}}\right)$, $\mu_{\kappa }=\mu_{0}\frac{\omega \omega_{m}}{\omega_{0}^{2}-\omega^{2}}$ with parameters as *ω*_0_ = 2*πγH*_0_, *ω*_*m*_ = 2*πγ*(4*πm*_*s*_), *γ* = 2.8×10^6^(rads^−1^G^−1^), and 4*πm*_*s*_ = 1780 G. For frequency 4.28 GHz, the dependence of *μ*_*r*_ and *μ*_*κ*_ as well as *μ*_*κ*_/*μ*_*r*_ on *H*_0_ is shown in Figure 2.

**Figure 3:**
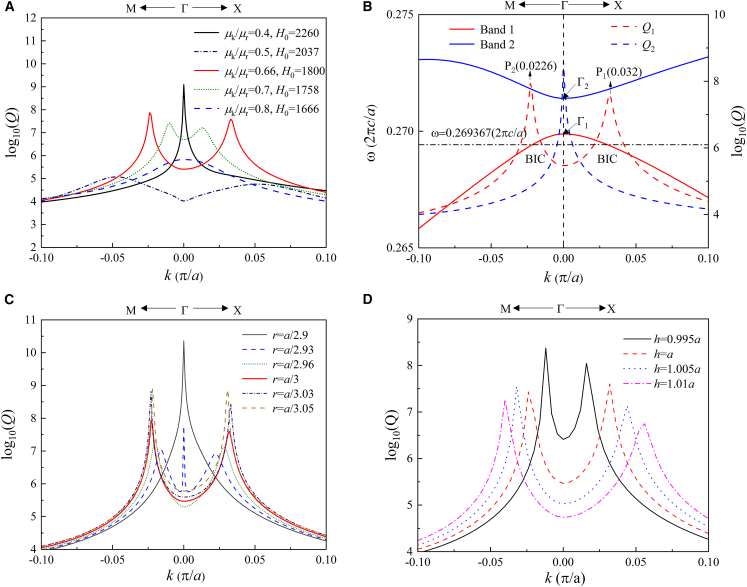
Band properties of the PhC slab with different parameters (A) The *Q* factors of band 1 with different *H*_0_. (B) Bands and *Q* factors with *H*_0_ = 1800G. (C) The *Q* factors of band 1 with *h* = *a* and different *r*. (D) The *Q* factors of band 1 with *r* = *a*/3 and different *h*.

Because the bandwidth in this study is very small, the dispersion effect of $\tilde{\mu }$ can be neglected. There are three key factors determining the results: *H*_0_, the hole radius *r,* and the slab thickness *h*. For *r* = *a*/3 and *h* = *a*, we firstly study the evolution of *Q* factors along band 1 from the anti-crossing bands with the increase in *μ*_*k*_/*μ*_*r*_. When increasing *μ*_*k*_/*μ*_*r*_, the *Q* peak undergoes the evolution from a single peak at Γ to double side peaks, and finally vanishes. In Figure 3A we focus on the result of *H*_0_ = 1800 Gauss, and plot the two anti-crossing bands and the *Q* factors along them in Figure 3B. Compared with Figure 1B, the frequency range is decreased, but it still has the anti-crossing shape due to the coupling effect. An important difference is the distribution of *Q* factors on the bands. A *Q* peak has been transformed from Γ_1_ to Γ_2_. On band 1, two *Q* peaks have left the center Γ point and occurred at P_1_ and P_2_ on the paths of ΓX and ΓM, respectively. Because P_2_ is in the direction of ΓM, its actual length to the Γ_1_ point is $\sqrt{2}\times 0.0226=0.032\left(\pi /a\right)$, which is equal to the length from P_1_ to Γ_1_. Keeping *H*_0_ and *h* invariant, the evolution of *Q* factors along band 1 with different *r* is shown in Figure 3C. For *r* = *a*/2.9, only a single *Q* peak occurs at Γ_1_. With the decrease of *r*, the central *Q* peak drops and the two side *Q* peaks rise. When *r* = *a*/2.96, the central *Q* peak disappears and only two side *Q* peaks remain. When *r* = *a*/3.05, the two side *Q* peaks reach their maximum peak value. With further decreasing of *r*, the two side *Q* peaks will diffuse and the total band 1 will become close to the waveguide guide modes. Keeping *r* = *a*/3 and *H*_0_ invariant, the evolution of *Q* values along band 1 with different *h* is shown in Figure 3D. The two side *Q* peaks are very sensitive to *h*. Only a small change of *h* will lead to a large change in the shape of the two *Q* peaks. With the increase of *h*, the interval between two *Q* peaks increases, but the peak heights decrease.

### Formation of the ring qBIC

**Figure 4:**
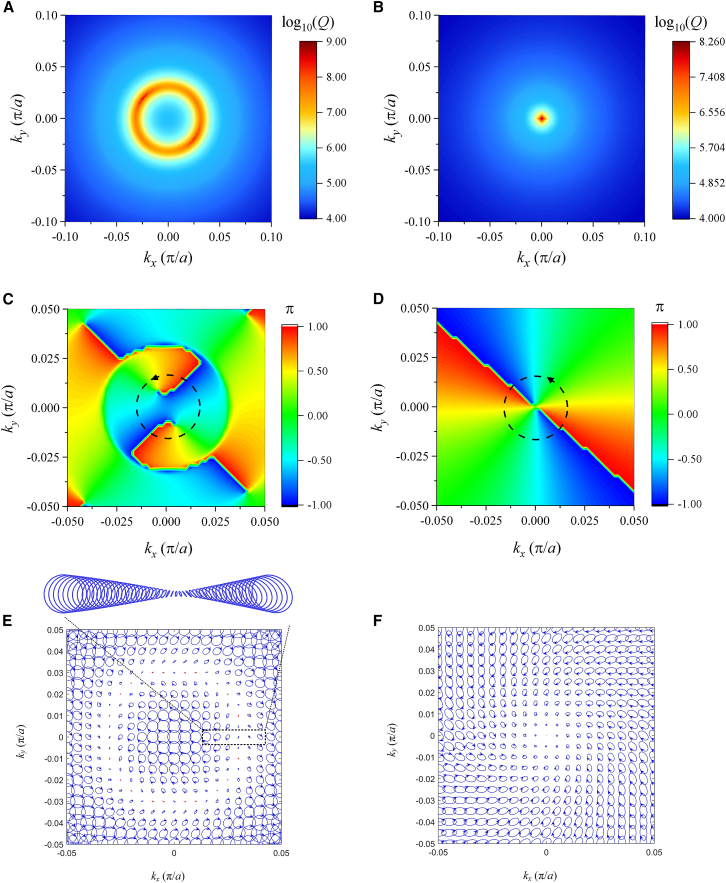
The 2D distributions of *Q* values, phases, and far-field polarizations for the two anti-crossing bands of the PhC slab with *H*_0_ = 1800G (A) *Q* values of band 1. (B) *Q* values of band 2. (C) Phases of band 1. (D) Phases of band 2. (E) Far-field polarizations of band 1. (F) Far-field polarizations of band 2.

Two questions arise: Are the three mode points Γ_2_, P_1,_ and P_2_ BIC? And how do the *Q* peaks occur in different scanning directions except for ΓX and ΓM? To answer these questions, the 2D distributions of *Q* values, phases, and far-field polarizations for the two bands have to be plotted in Figure 4 which shows a perfect mutual corroboration among them and a clear contrast between the two bands. In the following, the field polarization vectors are taken from the two components of *H*_x_ and *H*_y_. Because of the breaking of time-reversal symmetry from the magneto-optical effect, the far-fields become elliptical polarizations. The reason is analyzed in method details. We can still use Equation 1 to calculate the topological charge, but the azimuthal angles should be the azimuthal angles of an ellipse with $\phi =\arctan\frac{2\left|c_{x}\right|\left|c_{y}\right|\cos\left(\varphi_{x}-\varphi_{y}\right)}{{\left|c_{x}\right|}^{2}-{\left|c_{y}\right|}^{2}}$ , where *c*_*x*_(**k**) and *c*_*y*_(**k**) are the two components of elliptical polarizations, and *φ*_*x*_ and *φ*_*y*_ are phase angles of *c*_*x*_(**k**) and *c*_*y*_(**k**), respectively. For band 1, the *Q* peaks form a ring with a radius *k* = 0.0336 (*π*/*a*) in *k*-space in Figure 4A, correspondingly, in Figure 4C, the phases form interesting double-layer vortexes. The *Q* peaks in Figure 3A just occur at the double-layer vortex boundary. According to Equation 1, the two vortexes have the same topological charges *q* = −2. In Figure 4C, the phases at the ring interface of two vortices become discontinuous, so that the polarizations at the ring boundary in Figure 4C cannot determine their directions. The polarization distribution for band 1 is shown in Figure 4E in which the polarization at Γ_1_ is close to circular polarization. As the distance from Γ_1_ to the ring boundary in the *k*-space increases, the amplitudes of modes decrease to the minimum value and the ellipticities of modes are transformed from approximate circular polarization to linear polarization (see the enlarged figure). When the distance is larger than the ring boundary, the amplitudes and ellipticities of modes change in a reversed fashion. For band 2, the *Q* peak only occurs at Γ_2_ in Figure 4B. The phase distribution for band 2 is shown in Figure 4D, in which the phase singular point also occurs at the *Q* peak position, and the vortex has also topological charge *q* = −2. The polarization distribution is shown in Figure 4F, in which all the polarization vectors form a clear vortex with a vacant singular point at the center, Γ_2_. Considering the *Q* factor, phase singular point, and far-field polarization distribution, the mode of band 2 at Γ_2_ is undoubtedly BIC, and the modes of band 1 at the *Q* ring are all quasi(q)-BICs.

### Analysis of the ring qBIC formation

**Figure 5:**
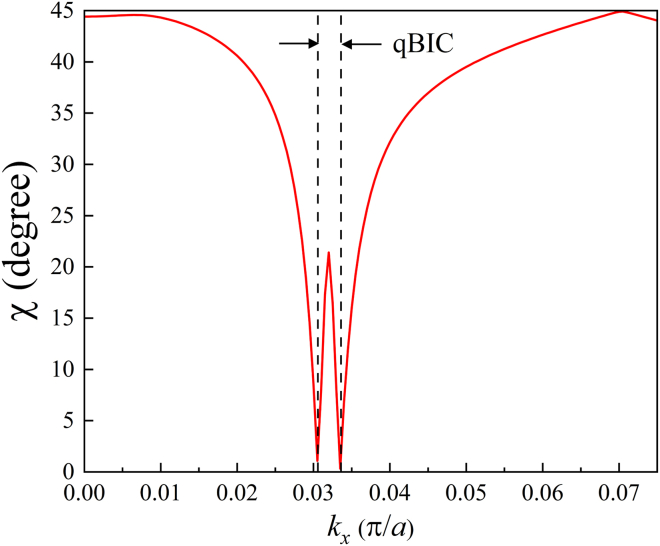
The ellipticities of band 1 along *k*_*x*_ direction

The ring qBICs, to our knowledge, are the special discovery of continuous qBICs, which make a clear contrast to general discrete BICs. The reason for forming the unique ring qBICs is qualitatively analyzed from TCMT in the method details. The far-fields within the ring provide rich polarization states. To account for them, we plot the ellipticities *χ* = arc*tan*(*a*_2_/*a*_1_) along *k*_*x*_ direction across the ring boundary, which is shown in Figure 5, where *a*_1_ and *a*_2_ are the lengths of the long axis and short axis of the ellipses, respectively. Two minimum values, 1 and 0 degrees of *χ* occur at *k*_*x*_ = 0.0306 and *k*_*x*_ = 0.0336, that is, the inner and outer boundaries of the ring, respectively. At *k* = 0, the ring center, *χ* is equal to 44.5° close to circular polarization. Within the *Q* ring, according to the values of *χ*, the far-fields undergo most potential polarization states. With the breaking of time-reversal symmetry and the holding of spatial symmetry, the PhC slab can still achieve BICs, but the far-field polarizations have been transformed from linear polarizations to general elliptical polarizations. The rich polarization states can be used to enhance the multiplexing capacity of information coding in optical communication systems. Thus, the formation of ring qBICs is the result of the breaking of time symmetry.

**Figure 6:**
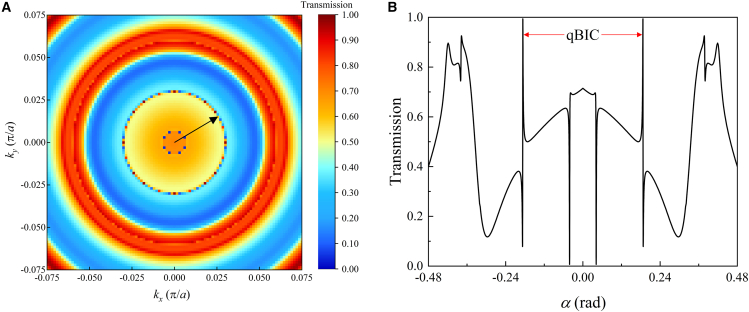
The transmission property of the PhC slab with *H*_0_ = 1800G and *ω* = 0.2693 (2*πc*/*a*) (A) 2D transmission spectrum in *k* space. (B) 1D transmission spectrum along *k*_*x*_ direction.

We focus on the ring qBICs and study their transport features through the frequency-domain simulations based on the COMSOL model with ports and periodic boundaries. The frequency is fixed at *ω* = 0.2693 (2π*c*/*a*), which is just at the ring boundary (see Figure 2A). Through scanning the incident angle $\alpha =\arcsin\left(\sqrt{k_{x}^{2}+k_{y}^{2}}/k\right)$ where *k* = *n*_*eff*_*ω*/*c* is the wave number within the slab and *n*_*eff*_ is the effective refractive index of the PhC slab. The 2D map of transmittance with *k*_*x*_ and *k*_*y*_ is shown in Figure 6A. A clear peak circle (denoted by an arrow) is found at the radius 0.032 (*π*/*a*) corresponding to the ring Q peaks in Figure 4A. For a detailed observation, along *k*_*x*_ direction of Figure 6A, we plot the transmittance as a 1D plot in Figure 6B. Two narrow Fano peaks occur just at the ring *Q* peak, denoting the qBICs. We also notice that there are other Fano resonance peaks with a small radius in Figure 6A and a small angle in Figure 6B, respectively. We think that they are result of the coupling of grating mode with high-*Q* and cavity resonance with low-Q, and they are not relevant to QBIC.

### Effect of magnetic field on the ring qBIC

**Figure 7:**
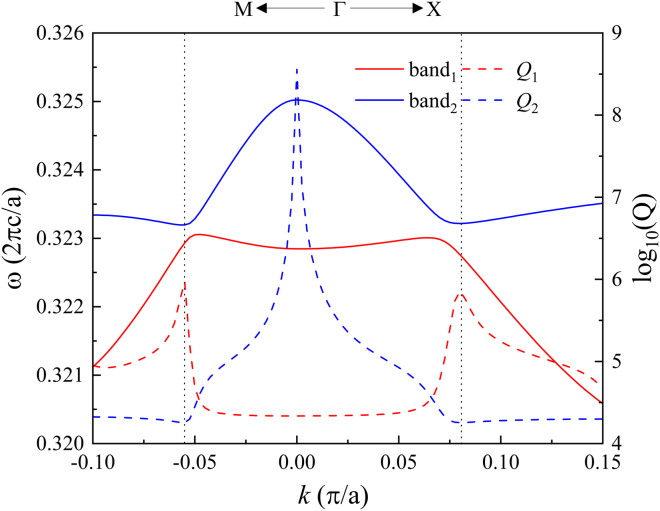
Frequency bands (left *y* axis) and *Q* factors (right *y* axis) of the PhC slab with *H*_0_ = 1600G

We studied the effect of magnetic fields on the ring qBIC. We found that *H*_0_ = 1600 Gauss, leads to *μ*_*r*_ = 14*μ*_0_, *μ*_*κ*_ = 12.4*μ*_0_. The magneto-optical effect is dependent on the ratio of *μ*_*κ*_/*μ*_*r*_. The current ratio of 0.886 for 1600G is larger than 0.662 for 1800G. The two frequency bands and corresponding *Q* values are plotted in Figure 7.

**Figure 8:**
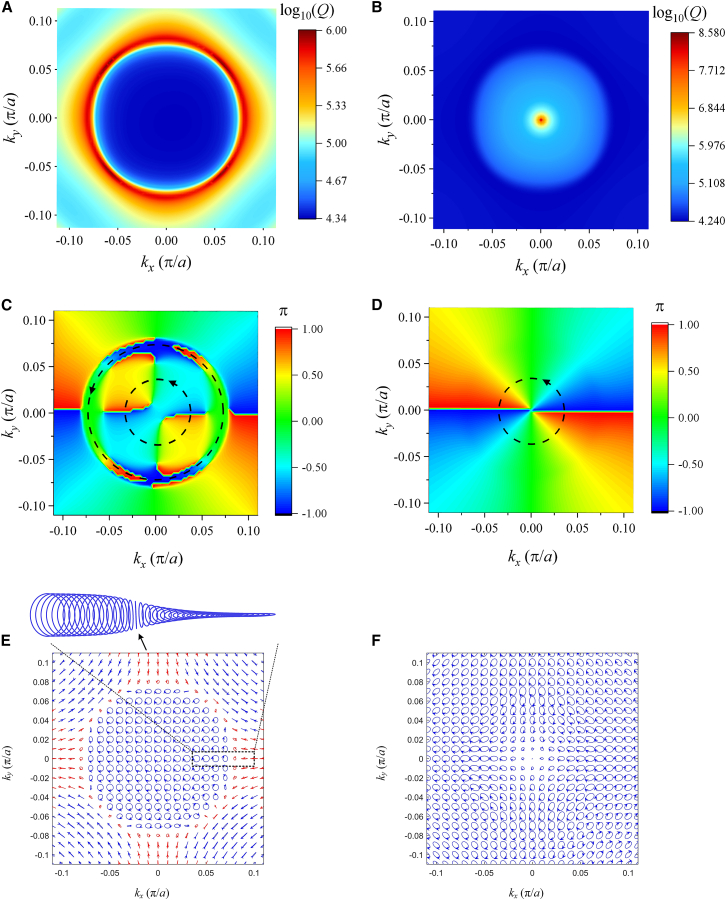
The 2D distributions of *Q* values, phases, and far-field features for two anti-crossing bands of the PhC slab under *H*_0_ = 1600G (A) *Q* factors of band 1. (B) *Q* factors of band 2. (C) Phases of band 1. (D) Phases of band 2. (E) Far-field polarizations of band 1. The arrow denotes a linear polarization. (F) Far-field polarizations of band 2.

**Figure 9:**
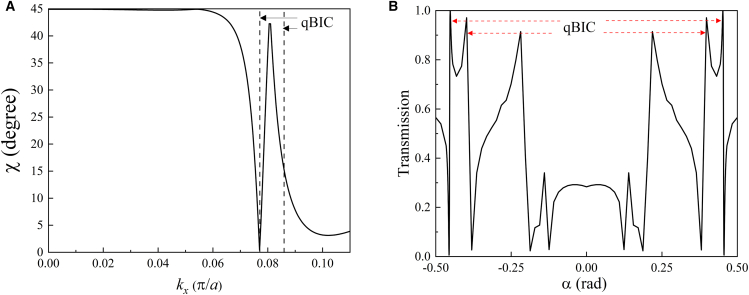
Representations of qBICs in two ways (A) The distribution of *χ* values along the *k*_*x*_ axis. (B) The 1D angle transmittance spectrum along the *k*_*x*_ direction with *ω* = 0.32288 (2*πc*/*a*).

Compared with Figure 2A, the anti-crossing centers have deviated from the Γ_1_ point and symmetrically occurred in the *k*-space. Close to the anti-crossing point, a pair of *Q* peaks and valleys occur on bands 1 and 2 with *k* = 0.081 (*π*/*a*) in the Γ-*x* direction, respectively, which correspond to a pair of QBICs and resonance states. The two vertical dotted lines denote the correspondence relation between the *Q* peak and anti-crossing band centers. The distributions of 2D *Q* values, phases, and far-field polarizations are plotted in Figure 8, which shows a mutual corroboration and a clear contrast for the two bands. The reason is discussed in the method details. For band 1, a ring of *Q* peak distribution in Figure 8A still occurs, but with a larger radius *k* = 0.08 (*π*/*a*) than that in Figure 4A. Correspondingly, the phases have an interesting distribution in Figure 8C. There is an overlap of two vortexes. One vortex occupies (small circle dashed line) the whole *k*-space with a topological charge *q* = 2. The other vortex (large circle dashed line) has a ring shape with a small width from 0.077 to 0.085 (*π*/*a*) (The *Q* peak just occurs at the center of the two boundaries of the ring) and the same topological charge *q* = 2. The phase discontinuity between the two vortex interfaces leads to the formation of ring qBIC. The polarization distribution in Figure 8E has a sharp contrast between the inner and outer spaces of the *Q* ring. Inside the ring (*k* < 0.081 (*π*/*a*)), most modes are circular polarizations with the same rotation direction, while outside the ring, all the modes are close to linear polarization. For band 2, the *Q* peak only occurs at Γ_2_ in Figure 8B. Correspondingly, the phase singular point also occurs at the *Q* peak position in Figure 8D, and the vortex has also topological charge *q* = 2. In Figure 8F, all the polarization vectors form a clear vortex with a vacant point at the center, Γ_2_. To make a detailed observation of the polarization distribution in Figure 8E, we plot the angle values of *χ* = arc *tan*(*a*_2_/*a*_1_) along *k*_*x*_ direction in Figure 9A. From *k*_*x*_ = 0 to *k*_*x*_ = 0.055(*π*/*a*), *χ* is always 45° (ideal circle). The wide flat spectrum of *χ* in the ring *k*-space means that the structure can output a circular polarization field in a large solid angle. When *k*_*x*_ is larger than 0.055 (*π*/*a*), *χ* quickly drops, and at *k*_*x*_ = 0.077 (*π*/*a*), just the inner boundary of the ring phase vortex, it reaches 0° (ideal line). Outside the inner *Q* ring (*k*_*x*_ > 0.077 (*π*/*a*)), *χ* undergoes an initial rise and then drops to small values. Through the localized amplification of Figure 8C, we find that a line polarization just occurs at the inner boundary of the vortex ring corresponding to the zero value of *χ*. The distribution of *χ* values in Figure 9A is well in agreement with the distribution of polarization fields in Figure 8E. We can conclude that the modes at the two boundaries of the *Q* ring on band 1 are qBICs, and the *Q* singular point on band 2 forms a BIC. Comparing the results of Figures 4 and 8, we find that the change of the external magnetic field has little effect on the band with a single BIC, but results in a strong influence on the band with the ring qBICs through changing the ring radius and polarization distribution.

The ring qBICs can be verified through the transport spectrum. The 1D plot of transmittance for *ω* = 0.32288 (2*πc*/*a*) (corresponding to the *Q* peak of band 1 in Figure 6 along *k*_*x*_ direction is shown in Figure 9B. Two pairs of Fano transmission peaks are symmetrically located at the two sides of Γ (*α* = 0) as the two dashed arrow points. The Fano peaks just correspond to the two interfaces of the phase ring in Figure 8C.

## Discussion

Controlling polarization is very important in a lot of fields, such as 3D imaging,^30^ optical communications,^31^ quantum optics.^32^^,^^33^ Compared with linear polarization, elliptical polarization states provide additional degrees of freedom, such as ellipticity, rotation direction, and azimuth angle. Thus, for information encoding, constructing multiple polarization states at once time becomes more important. In the current study, the ring qBICs in Figure 4E have resulted in all potential ellipticities for the elliptical polarizations within a ring *k*-space. The infinite density of states will potentially find important applications in the polarization encoding of photons on chips. In Figure 8E, the circular polarization modes occupy a large k-space, which can lead to a far-field circular polarization radiation within a large solid angle. It can improve the applicability of circular polarization devices in actual complex environments, especially for dynamic or multi-angle scenarios (satellite communications, mobile terminals, and other communication fields), such as unmanned aerial vehicles, vehicle radar, and other dynamic platforms that need to receive signals from different directions. The wide-angle spectrum characteristics can avoid frequent adjustment of device altitude. Although this is a theoretical study, the single-layer PhC slab with air holes is easily processed in an experiment. The only potential challenge is the dispersion effect of the material, which may decrease the Q value of qBICs. But the dispersion effect does not influence the formation of ring qBIC.

This work finds a form of qBIC with a continuous ring distribution from a magneto-optical PhC slab. Unlike general BIC systems in which the far-field polarizations are linear, the far-field polarizations with the ring qBICs are elliptical polarizations with all potential values of ellipticities. The results have proposed a unique formation mechanism of BIC. The unique form of qBICs may lead to applications in producing circular polarization light sources with large space angle spectra, designing spatial filters and encoding systems with many more degrees of freedom.

### Limitations of the study

In this study, we have not considered the dispersion effect of the material, which may decrease the *Q* value of qBICs.

## Resource availability

### Lead contact

Requests for further information and resources should be directed to and will be fulfilled by the lead contact, Sailing He (sailing@kth.se).

### Materials availability

This study did not generate new unique reagents.

### Data and code availability

• Data reported in this article will be shared by the lead contact upon request.
• This article does not report original code.
• Any additional information required to reanalyze the data reported in this article is available from the lead contact upon request.

## Acknowledgments

The work is partially supported by the 10.13039/501100001809Natural Science Foundation of China (W2412107), the 10.13039/501100012166National Key Research and Development Program of China (2022YFB2804100, 2022YFC3601003, 2022YFC2010003), and the “Pioneer” and “Leading Goose” R&D Program of Zhejiang Province (No. 2025C02159). The authors are grateful to Dr. Julian Evans of Zhejiang University for valuable discussions.

## Author contributions

The first author, Yuntuan Fang, has made the design idea and the writing of the article. Fan Bu has made the simulation and the process of data. Sailing, He has directed the total study.

## Declaration of interests

The authors declare no competing interests.

## STAR★Methods

### Key resources table

**Table:** 

REAGENT or RESOURCE	SOURCE	IDENTIFIER
**Software and algorithms**		

COMSOL Multiphysics 6.2	COMSOL Co., Ltd.	https://cn.comsol.com/
MATLAB 2024a	MathwWorks	https://www.mathworks.com/products/matlab.html
Origin 2022	Origin Lab	https://www.originlab.com/

### Experimental model and study participant details

Our study does not use experimental models typical in the life sciences.

### Method details

#### Structure design and property analysis

Numerical calculations and simulations of photonic crystal slab structures are performed using the commercial software COMSOL physics with the radio frequency module (electromagnetic waves, frequency domain). The specific model structure design is referenced in Design of the model and appearance of two Q-peaks. MATLAB is used to perform data calculation and processing on the calculated *H*_*x*_ and *H*_*y*_ to obtain phase and polarization properties. In the simulation of qBIC, the structure is periodically extended in the *x* and *y* directions, with periodic boundary conditions set around, ports set above and below, and an incident beam at a certain angle added to one of the ports. The electric field components are perpendicular or parallel to the *xy* plane, respectively. By using the frequency at the center of the Q peak and scanning the wave vectors *k*_*x*_ and *k*_*y*_, Fano resonance can be obtained.

#### Formation of elliptical polarization

Using the Bloch theorem for photonic crystals,^1^ we write the (electric or magnetic) field of a resonance as A(ρ,*z*) = *e*^*i*k·ρ^u_k_(ρ,*z*), where $k=k_{x}\hat{x}+k_{y}\hat{y}$ is the two-dimensional wave vector, $\rho =x\hat{x}+y\hat{y}$ is the in-plane coordinate, u_k_(ρ,*z*) is a periodic function in ρ, and *z* is the normal direction to the slab. For states above the light line (resonances), and wavelengths below the diffraction limit, the only nonzero propagating-wave amplitudes are the zero-order (constant in-plane) Fourier coefficients of u_k_, given by $c\left(k\right)=c_{x}\left(k\right)\hat{x}+c_{y}\left(k\right)\hat{y}$ with $c_{x}\left(k\right)=\hat{x}\cdot \left\langle u\left(k\right)\right\rangle$ and $c_{y}\left(k\right)=\hat{y}\cdot \left\langle u\left(k\right)\right\rangle$. The brackets represent the spatial average value of the electric field in the x-y plane of any unit in the far-field area outside the slab. c(k) is the projection of ⟨u(k)⟩ onto the *x*-*y* plane; it corresponds to the polarization vector of the resonance in the far field. When the Hermitian system is invariant under the operation $C_{2}^{z}T$, c(k) = c^∗^(k). In this case, *c*_x_ and *c*_y_ should be real numbers simultaneously, that is the far field is linearly polarized.^1^ Here $C_{2}^{z}$ is a 180° rotation operator around the z axis, and *T* is the time reversal operator. However, if the time-reversal symmetry is broken for the PC slab, c(k)≠c^∗^(k), then *c*_x_ and *c*_y_ have to be complex numbers, that is c(k) is an elliptical polarization. The breaking of time-reversal symmetry often occurs when the PC slab is made of magnetic medium under external magnetic field.

#### Derivation of the formation of qBIC using TCMT

The PhC slab with time and space inversion symmetry has resulted in a pair of BIC and resonance state which occur at the two Γ points on two anti-crossing bands. But under the external magnetic field, the resonant state has been transformed into a ring of quasi-BICs, while BIC is kept. In this section, we use time-domain coupled mode theory (TCMT) to qualitatively analyze the forming reasons of BIC and ring qBICs.

For a plane waveguide, there are only two guide mode bands degenerated at *k* = 0, see Figure S1A. When the plane waveguide includes periodicity and becomes a PhC slab, the two guided modes are coupled with the photonic modes of PhC slab. Strong coupling between two radiation channels of the two hybrid modes induces constructive and destructive interference, and an anticrossing of the two angular dispersion lines. The former produces a leaky mode with strong radiation losses; the latter leads to a BIC with an infinite radiative *Q* factor. The leaky mode(resonance) and BIC are the two lifted modes at the anti-crossing band center (Figure S1B). TCMT describes the behavior of resonances in a system in the time domain. In the present case, the system involves two resonant modes with a near field coupling and a far (radiation) field coupling, and the latter via the 0^th^ order grating corresponding to an input/output port. The time evolution of the two-mode amplitude A^*T*^ = [*a*_1_,*a*_2_] can be described by *D* = [*d*_1_, *d*_2_] as

(Equation 3) $$\frac{dA}{dt}=-\left(i\Omega -B\right)A+D^{T}s$$

where $\Omega =\left(\begin{array}{cc} \omega_{1} & \kappa \\ \kappa & \omega_{2} \end{array}\right)$ in which *ω*_1_ and *ω*_2_ are the eigenfrequencies of the two resonant modes and *κ* is the near-field coupling coefficients. $B=\left(\begin{array}{cc} \gamma_{1} & \gamma_{12}\\ \gamma_{21} & \gamma_{2} \end{array}\right)$ is the attenuation matrix of the resonator in which *γ*_1_ and *γ*_2_ are the radiative losses of the resonant modes and the nondiagonal elements *γ*_12_ and *γ*_21_ are the far-field couplings. $\gamma_{12}=\gamma_{21}=\sqrt{\gamma_{1}\gamma_{2}}$ if the two resonances have the same even-symmetric modes, $D^{T}=\left[\begin{array}{l} d_{1}\\ d_{2} \end{array}\right]$ is the coupling coefficients matrix between the resonant modes and the ports. *s* is the incident wave. The Hamiltonian of the system at this point is:

(Equation 4) $$H=\Omega -iB=\left(\begin{array}{cc} \omega_{1} & \kappa \\ \kappa & \omega_{2} \end{array}\right)-i\left(\begin{array}{cc} \gamma_{1} & \gamma_{12}\\ \gamma_{21} & \gamma_{2} \end{array}\right)$$

The eigenfrequencies *ω* can be obtained through solving the following equation

(Equation 5) $$\left|\omega E-H\right|=\left|\begin{array}{cc} \omega -\left(\omega_{1}-i\gamma_{1}\right) & -\kappa +i\sqrt{\gamma_{1}\gamma_{2}}\\ -\kappa +i\sqrt{\gamma_{1}\gamma_{2}} & \omega -\left(\omega_{2}-i\gamma_{2}\right) \end{array}\right|=0$$

where E is the unit matrix. Setting the two solutions of the above Hamiltonian quantity as *ω*_*a*_ and *ω*_*b*_, we have

(Equation 6) $$\left\{ \begin{array}{l} \omega_{a}+\omega_{b}=\omega_{1}+\omega_{2}-i\left(\gamma_{1}+\gamma_{2}\right)\\ \omega_{a}\omega_{b}=\left(\omega_{1}-i\gamma_{1}\right)\left(\omega_{2}-i\gamma_{2}\right)+{\left(i\kappa +\sqrt{\gamma_{1}\gamma_{2}}\right)}^{2} \end{array}\right.$$

If there exists a BIC in the system, there must exist a real number solution for Equation 5. Let two real numbers *R*_1_ and *R*_2_ denote the real parts of *ω*_*a*_ and *ω*_*b*_, and *ω*_*b*_ = *R*_2_, we obtain *R*_1_+*R*_2_ = *ω*_1_+*ω*_2_ and

(Equation 7) $$\left\{ \begin{array}{l} \omega_{a}=R_{1}-i\left(\gamma_{1}+\gamma_{2}\right)\\ \omega_{b}=R_{2} \end{array}\right.$$

Combining Equation 7 an Equation 6 leads to

(Equation 8) $$R_{2}=\frac{\omega_{1}\gamma_{2}\omega_{2}\gamma_{1}-2\kappa \sqrt{\gamma_{1}\gamma_{2}}}{\left(\gamma_{1}\gamma_{2}\right)}$$

and

(Equation 9) $$\left\{ \begin{array}{l} R_{1}+R_{2}=\omega_{1}+\omega_{2}\\ R_{1}R_{2}=\omega_{1}\omega_{2}-\kappa^{2} \end{array}\right.$$

Thus, *R*_1_ and *R*_2_ are the solutions of the following equation:

(Equation 10) $$x^{2}-\left(\omega_{1}+\omega_{2}\right)x+\left(\omega_{1}\omega_{2}-\kappa^{2}\right)=0$$

with

(Equation 11) $$x_{\pm }=\frac{\left(\omega_{1}+\omega_{2}\right)\pm \sqrt{{\left(\omega_{1}+\omega_{2}\right)}^{2}-4\left(\omega_{1}\omega_{2}-\kappa^{2}\right)}}{2}$$

Since *R*_2_ is one of the solutions, we have

(Equation 12) $$x_{+}\;\text{or}\;x_{-}=\frac{\omega_{1}\gamma_{2}+\omega_{2}\gamma_{1}-2\kappa \sqrt{\gamma_{1}\gamma_{2}}}{\left(\gamma_{1}+\gamma_{2}\right)}$$

Equation 12 can be transformed into:

(Equation 13) $$\sqrt{{\left(\omega_{1}-\omega_{2}\right)}^{2}+4\kappa^{2}}\;or-\sqrt{{\left(\omega_{1}-\omega_{2}\right)}^{2}+4\kappa^{2}}=-\frac{\left(\omega_{1}-\omega_{2}\right)\left(\gamma_{1}-\gamma_{2}\right)+4\kappa \sqrt{\gamma_{1}\gamma_{2}}}{\left(\gamma_{1}+\gamma_{2}\right)}$$

Squaring both sides lead to

(Equation 14) $${\left[\kappa \left(\gamma_{1}-\gamma_{2}\right)-\sqrt{\gamma_{1}\gamma_{2}}\left(\omega_{1}-\omega_{2}\right)\right]}^{2}=0$$

So the condition for forming a BIC is

(Equation 15) $$\kappa \left(\gamma_{1}-\gamma_{2}\right)=\sqrt{\gamma_{1}\gamma_{2}}\left(\omega_{1}-\omega_{2}\right)$$

Taking Equation 15 into Equation 6 leads to

(Equation 16) $$\omega =\left\{ \begin{array}{l} \frac{1}{2}\left(\omega_{1}+\omega_{2}\right)+\frac{\kappa }{2}\left(\sqrt{\frac{\gamma_{1}}{\gamma_{2}}}+\sqrt{\frac{\gamma_{2}}{\gamma_{1}}}\right)-i\left(\gamma_{1}+\gamma_{2}\right)\\ \frac{1}{2}\left(\omega_{1}+\omega_{2}\right)-\frac{\kappa }{2}\left(\sqrt{\frac{\gamma_{1}}{\gamma_{2}}}+\sqrt{\frac{\gamma_{2}}{\gamma_{1}}}\right) \end{array}\right.$$

The first solution with an imaginary part is a resonant state corresponding to the constructive interference of the two radiation channels in Figure S1, while the second solution without any imaginary part is the BIC corresponding to the deconstructive interference of the two radiation channels in Figure S1. When *κ* > 0, the BIC and resonant state are on the low frequency band and high frequency band, respectively, and when *κ* < 0, the case is reversed.

#### Formation of qBIC under external magnetic field

According to Equation 15, the forming of BIC is dependent on resonance modes *ω*_1_ and *ω*_2_ which are also dependent on the optical thickness of structure, especially, the effective refractive index of structure. For the magnetic PhC slab under the external magnetic field, we can still use TCMT to analyze the formation of BIC, but the result becomes different. See Figure S2. For linear polarized light propagating through the PhC slab in z direction, it becomes a pair of circular polarizations with opposite rotation directions because of the Faraday effect. For the magnetic PhC slab, there are effective permittivity *ε*_*eff*_ and permeability ${\tilde{\mu }}_{eff}=\left[\begin{array}{ccc} \mu_{r-eff} & i\mu_{\kappa -eff} & 0\\ -i\mu_{\kappa -eff} & \mu_{r-eff} & 0\\ 0 & 0 & \mu_{0} \end{array}\right]$. The left circular polarization and right circular polarization have different refractive indexes which are denoted as $n^{-}=\sqrt{\varepsilon_{eff}\mu_{r-eff}}+\delta$ and $n^{+}=\sqrt{\varepsilon_{eff}\mu_{r-eff}}-\delta$, respectively, where *δ* is proportional to *μ*_*κ*-*eff*_. Because of different refractive indexes, the resonance modes *ω*_1_ and *ω*_2_ have two values correspond to only two circular polarization, respectively. At Γ_2_ of band 2 the radiation channels for both circular polarizations induce deconstructive interference leading to BIC, while at Γ_1_ of band 1 the radiation channels only for one circular polarization result in constructive interference forming a circular polarization (see Figure 3E of the main text). For a smaller magneto-optical effect in Figures 2, 3, and 4 of the main text, the difference of *n*^-^ and *n*^+^ will decrease as the modes on band 1deviate from the center point Γ_1_ so that another circular polarization add into the far-field radiation forming elliptical polarizations. The mode ellipticities decrease with the increasing of *k* values of modes. Along with the change of frequency and the vanishing of difference of *n*^-^ and *n*^+^ on band 1, at a value of *k*, the two radiation channels with *n*^-^ and *n*^+^ are merged into one and make a deconstructive interference with the other radiation channel from band 2, which leads to QBICs. The formation of QBICs is only dependent on the *k* value and does nothing with the symmetry of *k*-space. Thus, the QBICs with the same *k* values form a ring in *k*-space with a continuous distribution. Within the ring, the polarizations undergo an evolution from circular polarization to linear polarization. For a larger magneto-optical effect in Figure 6, different from Figure 2, the larger difference of *n*^-^ and *n*^+^ can remain in a larger *k* range. Thus, in a larger *k* range of band 1, only the radiation channels from one circular polarization take part in the interference with the other radiation channels from the modes on band 2. As a result, the strict deconstructive interference occurs at Γ_2_ leading to the BIC, and the constructive interference occupies a larger *k*-space of band 1 leading to the distribution of circular polarizations in a large *k*-space in Figure 7E of the main text. As *k* moves to the anti-crossing position, the radiation channels of modes with both circular polarizations on band 1 make deconstructive interference with the other radiation channels of modes on band 2, leading to QBIC ring. Only at the inner ring interface, both the two circular polarizations have been merged into a linear polarization so that the polarizations at the inner ring interface are ideal linear polarization.

### Quantification and statistical analysis

There are no quantification or statistical analyses to include in this study.

### Additional resources

We have no relevant resources.
