# Supplementary material for: Ring bound states in the continuum with broken time-reversal symmetry
Source: iScience. 2025 Nov 17;28(12):114103. doi: 10.1016/j.isci.2025.114103 (PMC12719180; doi:10.1016/j.isci.2025.114103)
Supplement: Document S1. Figures S1 and S2 [file mmc1.pdf]

iScience, Volume *28*

Supplemental information

Ring bound states in the continuum with broken time-reversal symmetry

Yun-tuan Fang, Fan Bu, and Sailing He

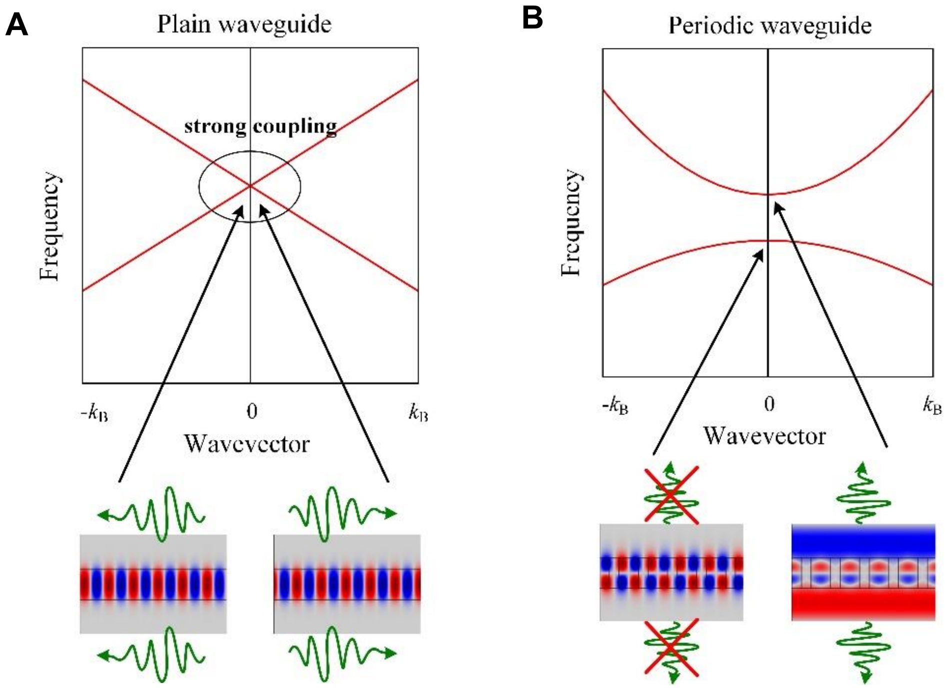

Figure S1. The BIC formation mechanism.

(A). A pair of leaky guided modes are degenerated at the momentum space origin.

(B). Two degenerated modes are strongly coupled when the slab becomes a PhC slab. The radiation channels from the lifted modes produce constructive and destructive interference. The former produces a leaky mode with strong radiation losses; the latter leads to a BIC with an infinite radiative Q factor.

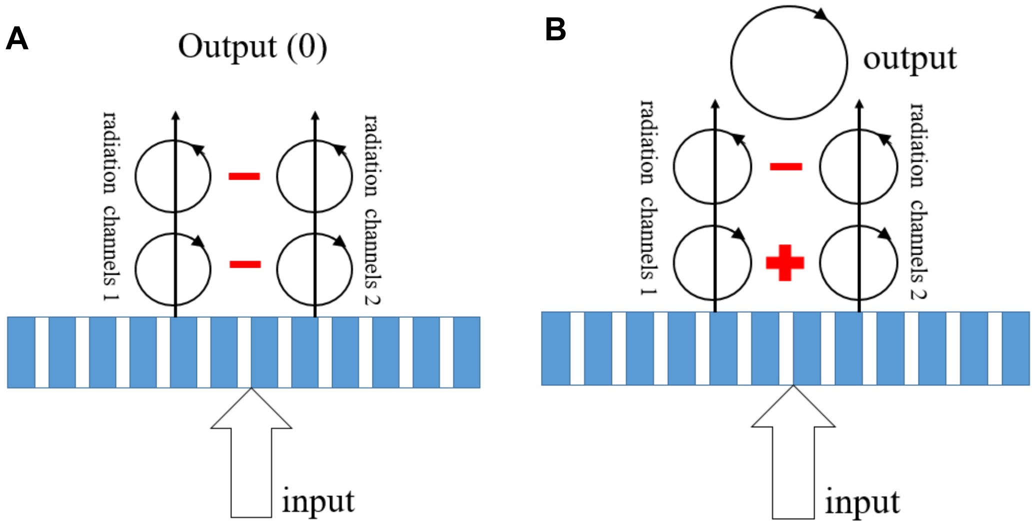

Figure S2. BIC and resonance formation mechanism from magneto-optical PhC slab.

(A) BIC.

(B) Resonance. The circle denotes the circular polarization. “+” and “-” denote constructive and deconstructive interference, respectively.
